# Determination of commissioning criteria for multileaf‐collimator, stereotactic radiosurgery treatments on Varian TrueBeam and Edge machines using a novel anthropomorphic phantom

**DOI:** 10.1002/acm2.13581

**Published:** 2022-03-15

**Authors:** Thomas A. D. Brown, Jessica M. Fagerstrom, Caleb Beck, Connor Holloway, Krista Burton, Darryl G. L. Kaurin, Saikanth Mahendra, Marcus Luckstead, Kayla Kielar, James Kerns

**Affiliations:** ^1^ Northwest Medical Physics Center Lynnwood Washington USA; ^2^ Varian Medical Systems Palo Alto California USA

**Keywords:** commissioning, phantom, stereotactic radiosurgery

## Abstract

An anthropomorphic phantom has been developed by Varian Medical Systems for commissioning multileaf‐collimator (MLC), stereotactic radiosurgery (SRS) treatments on Varian TrueBeam and Edge linear accelerators. Northwest Medical Physics Center (NMPC) has collected end‐to‐end data on these machines, at six independent clinical sites, to establish baseline dosimetric and geometric commissioning criteria for SRS measurements with this phantom. The Varian phantom is designed to accommodate four interchangeable target cassettes, each designed for a specific quality assurance function. End‐to‐end measurements utilized the phantom to verify the coincidence of treatment isocenter with a hidden target in a Winston‐Lutz cassette after localization using cone‐beam computed tomography (CBCT). Dose delivery to single target (2 cm) and single‐isocenter, multitarget (2 and 1 cm) geometries was verified using ionization chamber and EBT3 film cassettes. A nominal dose of 16 Gy was prescribed for each plan using a site's standard beam geometry for SRS cases. Measurements were performed with three Millennium and three high‐definition MLC machines at beam energies of 6‐MV and 10‐MV flattening‐filter‐free energies. Each clinical site followed a standardized procedure for phantom simulation, treatment planning, quality assurance, and treatment delivery. All treatment planning and delivery was performed using ARIA oncology information system and Eclipse treatment planning software. The isocenter measurements and irradiated film were analyzed using DoseLab quality assurance software; gamma criteria of 3%/1 mm, 3%/0.5 mm, and 2%/1 mm were applied for film analysis. Based on the data acquired in this work, the recommended commissioning criteria for end‐to‐end SRS measurements with the Varian phantom are as follows: coincidence of treatment isocenter and CBCT‐aligned hidden target < 1 mm, agreement of measured chamber dose with calculated dose ≤ 5%, and film gamma passing > 90% for gamma criteria of 3%/1 mm after DoseLab auto‐registration shifts ≤ 1 mm in any direction.

## 1 INTRODUCTION

Varian TrueBeam and Edge linear accelerators (Varian Medical Systems, Palo Alto, CA) have been shown to be excellent treatment platforms for stereotactic radiosurgery (SRS).^1^, ^2^, ^3^ Treatments may be delivered using stereotactic cones or one of two multileaf‐collimator (MLC) designs: the 120‐leaf Millennium MLC with a minimum leaf width of 0.5 cm, or the 120‐leaf high‐definition MLC (HDMLC) with a minimum leaf width of 0.25 cm.^4^ Stereotactic cones provide conical apertures as small as 0.4 cm and may be preferred for SRS treatments of small targets (<0.5 cm) such as the trigeminal nerve; however, studies have shown that the HDMLC can provide robust treatment delivery to targets of this size^5^, ^6^ and it has been utilized for the treatment of trigeminal neuralgia and essential tremors.^7^

End‐to‐end testing is a critical part of the commissioning process for SRS treatments and requires an evaluation of the geometric and dosimetric features of treatment delivery.^8^, ^9^, ^10^ There are a variety of anthropomorphic^11^, ^12^, ^13^ and non‐anthropomorphic phantoms^14^, ^15^ available to clinical physicists for end‐to‐end SRS measurements. One of the more commonly utilized devices is the anthropomorphic phantom developed by the Imaging and Radiation Oncology Core (IROC) Houston Quality Assurance Center.^11^ This is a thermoplastic head phantom containing a 1.9‐cm target that can be visualized with computed tomography (CT) or magnetic resonance imaging (MRI) for delineation during treatment planning. The phantom utilizes thermoluminescent detectors (TLDs) and radiochromic film for dosimetric and geometric treatment verification. This head phantom is owned by IROC Houston and sent to institutions that request an independent evaluation of their SRS treatment delivery. The end‐to‐end results obtained with the IROC phantom are often used for clinical trial credentialing in the United States. The STEEV and MAX‐HD 2.0 anthropomorphic phantoms^12^, ^13^ are examples of commercially available devices that offer a broader range of end‐to‐end tools. These phantoms utilize 1.6–3.0 cm targets for CT or MRI treatment planning and can accommodate a range of interchangeable dosimetric inserts including small‐volume chambers, radiochromic film, metal‐oxide‐semiconductor field‐effect transistors (MOSFETs), TLDs, optically stimulated luminescence detectors (OSLDs), and three‐dimensional (3D) gel. The phantoms also have inserts for Winston–Lutz delivery, CT/MRI/positron emission tomography (PET) fusion verification, and MRI distortion evaluation. The target size utilized in these anthropomorphic phantoms are broadly representative of many lesions targeted for SRS; a recent survey of cranial SRS in the United Kingdom indicated that single brain metastases ranging from 1 to 20 cm^3^ are the most commonly treated lesions.^12^, ^16^

Varian Medical Systems has now developed its own anthropomorphic phantom for commissioning MLC‐based, SRS treatments on TrueBeam and Edge linear accelerators. This phantom is specifically designed for performing end‐to‐end SRS measurements on these machines and has a similar range of tools as the STEEV and MAX‐HD 2.0 phantoms. It is designed to accommodate four interchangeable target cassettes that allow for CT‐MRI fusion verification, isocenter‐coincidence verification, on‐board imaging couch shift verification, ionization chamber measurements, and GAFchromic EBT3 radiochromic film (Ashland Advanced Materials, Bridgewater, NJ) measurements. Two spherical targets—1 and 2 cm in diameter—can be used for treatment planning. The phantom design was initially evaluated by Northwest Medical Physics Center (NMPC) and an MLC‐based, SRS end‐to‐end procedure for this phantom was developed for TrueBeam and Edge machines.^17^, ^18^ This procedure has now been performed for six linacs, at 6‐ and 10‐MV flattening filter‐free (FFF) energies, for the purposes of establishing baseline commissioning criteria for SRS measurements with this phantom. Data collection from multiple linacs using a standardized procedure permits the definition of a specific set of dosimetric and geometric commissioning criteria for prescribed tests on these machines, within the boundary conditions of published tolerances.^9^, ^19^ Moreover, these data are beneficial for establishing standardized SRS criteria and thresholds for gamma analyses of irradiated film obtained on these machines. Although the American Association of Physicists in Medicine (AAPM) Task‐Group 218 has established gamma criteria (3%/2 mm) and universal action (passing > 90%) and tolerance (passing > 95%) limits for non‐stereotactic intensity modulated radiation therapy (IMRT) treatments,^20^ there is no consistent metric presented in the literature for SRS treatments. Film irradiations with the IROC SRS phantom are analyzed using gamma criteria of 5%/3 mm; a global gamma passing rate of > 85% is used as the threshold for clinical trial credentialing.^21^ Dimitriadis et al.^12^ evaluated film irradiated in the STEEV phantom using a range of criteria from 2% to 5% and 1–2 mm for local and global gamma analyses. A mean global gamma passing rate of 96.7% for 2%/2 mm was reported for three successive SRS treatments on a TrueBeam using a seven‐field IMRT technique. Film irradiations for patient‐specific SRS quality assurance (QA)^22^, ^23^ have reported gamma analyses using criteria of 3%/2 mm, 3%/1 mm, and 2%/2 mm with dose thresholds of 10% and 20%. Xia et al. has applied Task Group‐218 action limits for patient‐specific QA of stereotactic treatments.^24^ These authors recommend stricter gamma criteria of 3%/1 mm for evaluating dose distributions with Delta4 (ScandiDos, Uppsala, Sweden), Portal Dosimetry (Varian Medical Systems, Palo Alto, CA), and the SRS MapCHECK (Sun Nuclear, Melbourne, Fl).

This study provides an overview of the Varian phantom design, a description of the end‐to‐end procedure, and a detailed analysis of the end‐to‐end data acquired across the six linacs. Recommended commissioning criteria for SRS measurements performed with this phantom are provided for TrueBeam and Edge machines consistent with published recommendations.^9^, ^19^

## 2 MATERIALS AND METHODS

### 2.1 Phantom design

**FIGURE 1:**
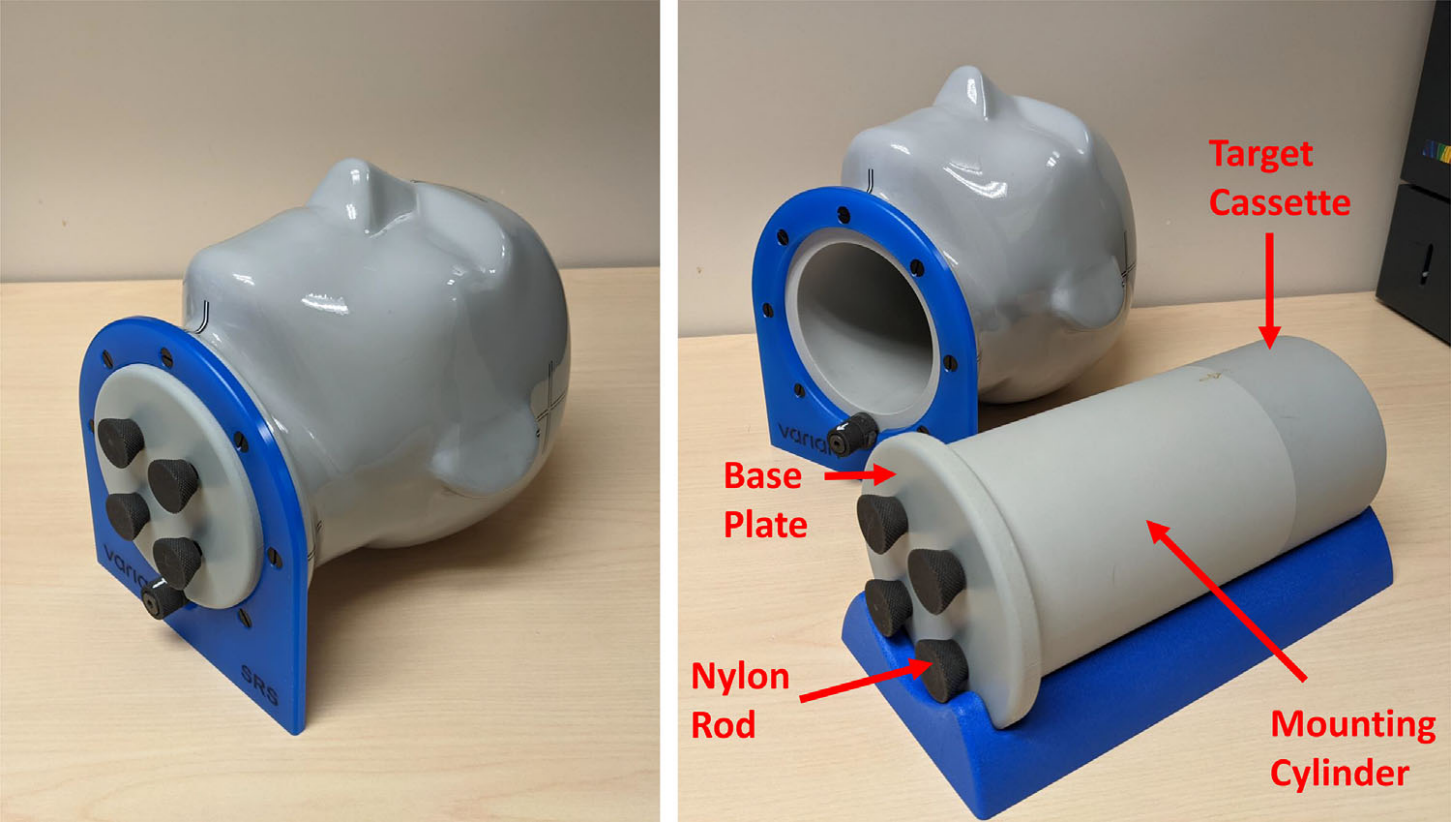
The Varian stereotactic radiosurgery (SRS) phantom. The phantom head contains a cylindrical cavity that allows for one of four target cassettes to be inserted for quality assurance measurements. The target cassette is attached to a mounting cylinder and base plate using four threaded nylon rods. There is small cylindrical cavity that runs through the base plate and mounting cylinder (not visible in figure); this allows for insertion of an ionization chamber into a target cassette

The Varian SRS phantom is shown in Figure 1; it is designed as a simplified geometric representation of a human head and measures approximately 18.5 × 26.5 × 22.7 cm. It comprises a 0.6‐cm thick, bone skull‐equivalent material encapsulated within a soft‐tissue equivalent material. Both materials are proprietary epoxy resins; however, CT images of the phantom indicate an average Hounsfield unit (HU) of approximately 850 and 30 for these materials, respectively. The phantom contains a cylindrical cavity that allows for one of four target cassettes to be inserted for geometric or dosimetric QA measurements. A target cassette is attached to a mounting cylinder and base plate using four threaded nylon rods; this target assembly is inserted into the phantom and secured in place using a rotating latch.

**FIGURE 2:**
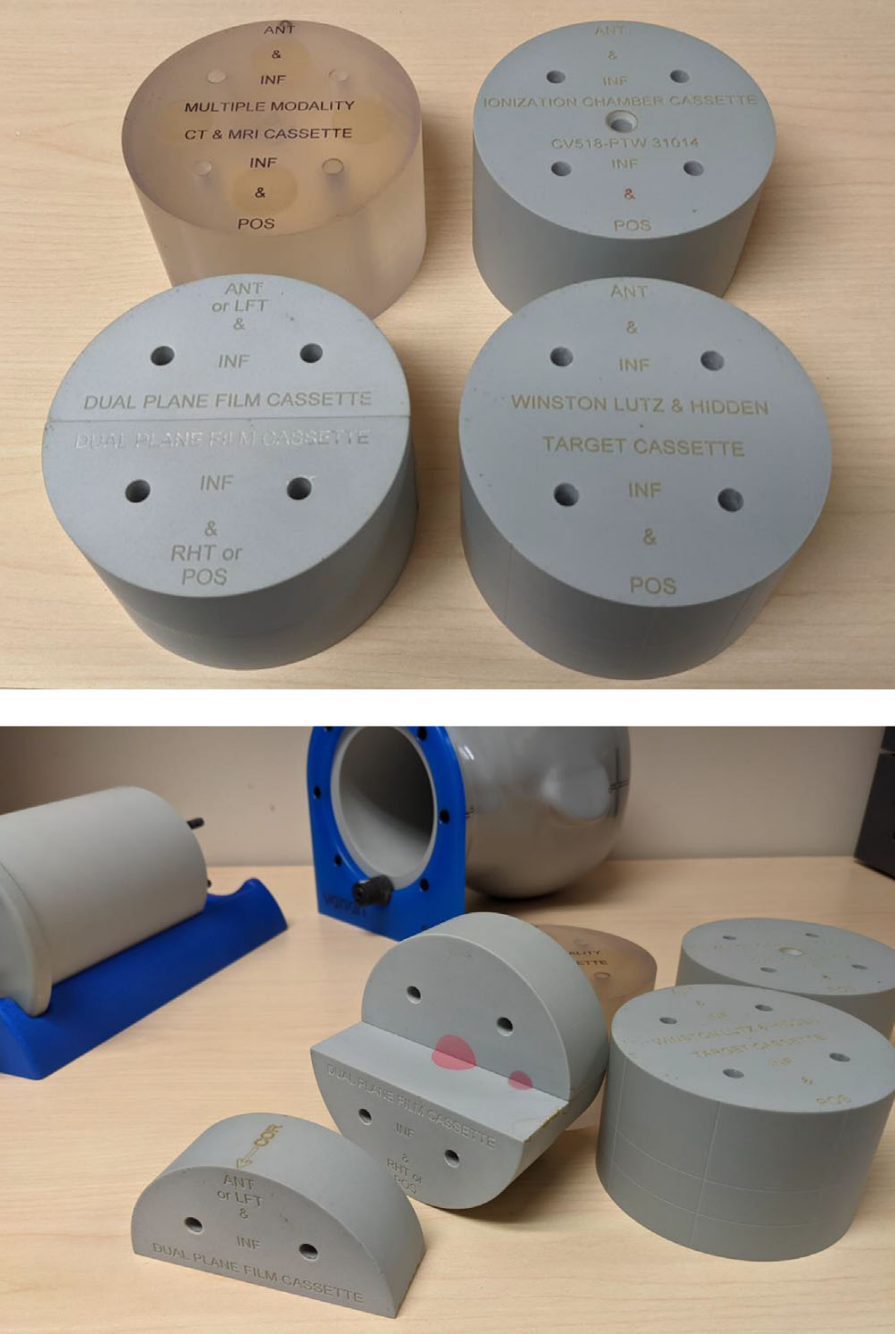
Target cassettes used for dosimetric and geometric quality assurance. (Top) Four types of target cassette, clockwise starting upper left: multiple‐modality computed tomography (CT) and magnetic resonance imaging (MRI), ionization chamber, Winston–Lutz/hidden target, and dual‐plane film cassettes. (Bottom) One of the four segments that comprises the film cassette has been removed to show the contrast‐enhanced (pink) targets used for planning

**FIGURE 3:**
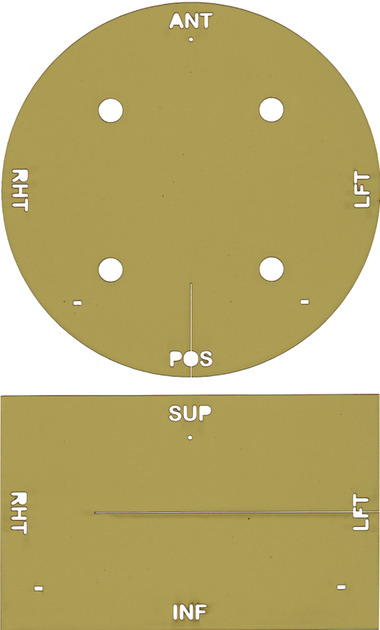
Precision‐cut pieces of EBT3 film designed for placement in the phantom film cassette. The film pieces shown above are designed for dose measurement in the axial (top) and coronal (bottom) planes. Each piece of film contains a small circular punch, located just below the ANT/SUP label, and two rectangular punches. These holes provide well‐defined fiducial points for translational and rotational registration during the analysis of the irradiated film

Each target cassette is a cylinder of radius 5 cm and length 6.3 cm and is designed for a specific QA function. Figure 2 shows the four types of target cassette. The Winston–Lutz/hidden target cassette contains two 0.8‐cm brass balls; one ball is embedded at the center of the cassette and the other is offset from center at distances of 2.5, 3.5, and 1.5 cm in the phantom's left, posterior, and superior directions, respectively. The ionization chamber cassette contains a 2 cm, 5% contrast‐enhanced target located at its center. It contains an internal cavity designed to accommodate a user‐specified chamber appropriate for SRS measurements. In this work, the cavity was drilled to accommodate a PTW pinpoint (0.015 cc) chamber (PTW, Freiburg, Germany). The dual‐plane film cassette also contains a 2 cm, 5% contrast‐enhanced target at its center as well as a second contrast‐enhanced target, 1 cm in diameter, located 3‐cm lateral from the center. The film cassette consists of four segments designed to accommodate two interlocking, orthogonal pieces of EBT3 film that can be mounted in the axial and sagittal or coronal planes. Each piece of film is precision‐cut for the phantom. The film pieces contain orientation labels and three fiducial holes to aid rotational and translation registration during analysis of the irradiated film. Figure 3 illustrates the design of the film pieces. These three target cassettes comprise the same epoxy resin designed to simulate soft tissue in the surrounding phantom. The fourth target cassette, a multiple‐modality CT‐MRI cassette, comprises two polyurethane gels doped to provide contrasting MR signals. The cassette contains a 2‐cm target at its center and six 0.75‐cm fiducial markers located at the edges of the cassette that are designed to define the three primary imaging planes. Due to limited access to MRI machines, the CT‐MRI target cassette was not utilized in this work. However, CT/MRI registration accuracy for this cassette was verified during the design evaluation of the phantom.^17^

### 2.2 End‐to‐end procedure

End‐to‐end testing with the Varian SRS phantom was performed for six TrueBeam and Edge machines located at six independent clinical sites (one machine per site). Each clinical site has an active SRS program and follows published recommendations for routine SRS QA.^9^, ^19^ Testing was performed with three Millennium and three HDMLC linacs at beam energies of 6‐ and 10‐MV FFF. Each machine has a six degree‐of‐freedom robotic couch, except for one whose motion axes was limited to four degree‐of‐freedom. Each clinical site followed a standardized procedure for phantom simulation, treatment planning, QA, and treatment delivery. All treatment planning and delivery was performed using ARIA and Eclipse (Varian Medical Systems, Palo Alto, CA).

#### 2.2.1 Simulation

The phantom was simulated according to the SRS procedures of each clinical site. The phantom has a stable rest position and will remain stationary for small pitch and roll couch angles (<1^o^). However, except for two clinics, a frameless SRS mask was fabricated for phantom immobilization at each institution. Qfix Encompass (Qfix, Avondale, PA), Qfix Integrated Shim for Portrait S‐frame, and Brainlab masks (Brainlab, Munich, Germany) were used in this work. Three separate CT scans of the phantom were obtained at all sites; one scan each for the Winston–Lutz, ion chamber, and film target cassettes mounted inside the phantom. In each case, a CT slice thickness ≤ 1.25 mm was used for imaging with the standard SRS scanning protocols used at each site. A soft‐tissue equivalent chamber plug, with the same dimensions as the ionization chamber, was inserted into the chamber cavity for the phantom CT scan with the chamber cassette. Similarly, mock pieces of polyester film were mounted in the film cassette, in the axial and coronal planes, prior to the scan with this cassette.

#### 2.2.2 Treatment planning

Two types of treatment plans were designed for SRS treatment delivery at each site: a single‐target plan and a single‐isocenter, multitarget plan. Both types of plans were designed on the CT image set of the phantom containing the film cassette. For the single‐target plan, the 2‐cm target at the center of the cassette was contoured as a planning target volume (PTV) using a high‐accuracy segmentation option. For the multitarget plan, the 2‐ and 1‐cm targets in the film cassette were contoured as high‐accuracy PTVs. A nominal dose of 16 Gy in one fraction was prescribed for both plan types. This dose was chosen as a compromise between an acceptable stereotactic dose and the dose saturation threshold of EBT3 film (20 Gy^25^). Each clinical site adopted a field configuration and treatment technique commonly used for local SRS patient treatments. All sites utilized four to seven parasagittal RapidArc fields for both plans, except for one site that used four dynamic conformal arcs (DCAs) for the single‐target plan. Couch angles varied between 90^o^ and 270^o^ and were separated by 10^o^–45^o^. The isocenter location was consistent across all sites; it was defined at the center of the 2‐cm target for the single‐target plan and equidistant between the 2‐ and 1‐cm targets for the multitarget plan. Tables 1 and 2 show the planning constraints for both types of treatment plan. These constraints were the same irrespective of MLC type and were developed based on the planning practice of the first three test sites.^18^

**TABLE 1:** Planning constraints for the single‐target (2 cm) plan. Constraints are the same for plans designed with Millennium and HDMLC

Planning objective	Constraint
PTV D99	≥16 Gy
Maximum dose	<20 Gy
Ideal CI	1.00–1.10
Acceptable CI	1.11‐1.25

Abbreviations: CI, conformity index; HDMLC, high‐definition multileaf collimator; PTV, planning target volume.

**TABLE 2:** Planning constraints for the single‐isocenter, multitarget (2 and 1 cm) plan. Constraints are the same for plans designed with Millennium and HDMLC

Target (PTV)	Planning objective	Constraint
2 cm	PTV D99	≥16 Gy
	Ideal CI	1.00–1.10
	Acceptable CI	1.11–1.25
1 cm	PTV D99	≥16 Gy
	Ideal CI	1.00–1.20
	Acceptable CI	1.21–1.50
2 cm and 1 cm	Maximum dose	<20 Gy
	Dose bridge between PTVs	<8 Gy (desirable not mandatory)

Abbreviations: CI, conformity index; HDMLC, high‐definition multileaf collimator; PTV, planning target volume.

After completion of the single‐target plan, a second single‐target plan was created by copying the first plan onto the CT image set containing the ion chamber cassette and recalculating the plan for the same number of monitor units. Plans were developed separately for 6‐ and 10‐MV FFF. All plan calculations were performed using Analytical Anisotropic Algorithm (AAA v15.5 – 15.6) with a 1‐mm isotropic dose grid. A total of six plans were developed for each linac: three plans per beam energy comprising one multitarget plan, designed for film verification, and two single‐target plans designed for both ion chamber and film verification.

In addition to the above treatment plans, a Winston–Lutz plan was designed to verify the coincidence of treatment isocenter with a hidden target positioned with cone‐beam computed tomography (CBCT). The plan was designed on the CT image set containing the Winston–Lutz/hidden target cassette and comprised 14, 6‐MV FFF imaging fields encompassing the full range of gantry, collimator, and couch rotations, with field size (∼2 × 2 cm) defined by the MLC. Table 3 shows the gantry, collimator, and couch angle combinations used for the plan. Isocenter was placed at the center of the brass ball located on the longitudinal axis of the cassette. A CBCT setup field was included for ball (target) alignment. Aside from changes in MLC type, the same Winston–Lutz plan was used across all six machines.

**TABLE 3:** Gantry, collimator, and couch angle combinations used for the Winston–Lutz plan across all six linacs. The angles shown below are for an IEC‐61217 machine scale

Gantry angle (^o^)	Collimator angle (^o^)	Couch angle (^o^)
0	0	0
0	90	0
0	270	0
0	0	45
0	0	90
0	0	315
0	0	270
90	0	0
180	0	0
180	0	45
180	0	90
180	0	315
180	0	270
270	0	0

#### 2.2.3 Quality assurance

Prior to performing the film and ion chamber measurements, specific QA procedures were followed on the day of irradiation to ensure the accuracy of treatment delivery. The MLC leaf positions were re‐calibrated by re‐initializing the MLC on the TrueBeam/Edge machines. Imaging isocenter was also re‐calibrated using the IsoCal procedure for these machines.^26^ These re‐calibrations help ensure the accuracy of dose modulation and localization for SRS treatments. The beam geometry and mechanical systems of the linac were verified to be within manufacturer‐specified tolerances using Varian's Machine Performance Check tool.^27^, ^28^ The beam output at 6‐ and 10‐MV FFF was measured using a Farmer chamber in a 20.4 × 20.4 × 13.6 cm acrylic phantom at a depth of 3.5 cm. This setup is utilized by NMPC for monthly output QA; output results obtained from this setup are benchmarked annually against AAPM Task Group‐51 dose measurements and include a correction for the volume‐averaging effect seen for Farmer chambers in FFF beams.^29^, ^30^, ^31^ The coincidence of treatment isocenter with a hidden target was verified using the Winston–Lutz plan described in Section 2.2.2. The phantom, containing the Winston–Lutz cassette, was placed inside the mask fabricated at simulation and aligned to plan isocenter using the CBCT imaging field. The brass ball located on the longitudinal axis of the cassette was used as the hidden target for alignment. Although there were imaging artifacts associated with the brass, the ball was easily visualized on the CT and the CBCT by adjusting window and level. Two CBCTs were always performed; a second CBCT was performed to verify the phantom hadn't moved as a result of the couch shifts following the first CBCT. This imaging procedure was taken from the SRS patient workflow at one of the clinical sites. It's possible for the patient to slide on a six degree‐of‐freedom couch, even inside a mask, if the pitch and roll angles are adjusted.^32^ After CBCT alignment, the Winston–Lutz imaging fields were delivered without adjusting the phantom position. The resulting images were analyzed using DoseLab (Varian Medical Systems, Palo Alto, CA).

#### 2.2.4 Dose measurement and analysis

The phantom was aligned for the film and chamber measurements in the same way as the Winston–Lutz measurements. For the single‐target plan, the central 2‐cm target was used as the primary landmark for CBCT alignment. For the multitarget plan, both targets were used as landmarks for alignment.

The ion chamber measurements were performed using a PTW N31006 pinpoint chamber. The total measured charge was corrected using standard correction factors (*P*_TP,_ *P*_elec_, *P*_pol,_ *P*_ion_) and converted to dose using a calibration factor obtained from a cross calibration against an Accredited Dosimetry Calibration Laboratory (ADCL)‐calibrated Farmer chamber. The measured dose was adjusted for beam output using the Farmer chamber measurement in acrylic described in Section 2.2.3. Given the pinpoint chamber size and the field sizes (> 2 cm) used for treatment, a small‐field correction factor was not applied to these measurements.^33^ The adjusted measured dose was compared against the calculated point dose at isocenter in the Eclipse plan. This location is coincident with the center of the chamber cavity, assuming correct CBCT alignment and chamber insertion into the phantom.

Four of the clinical sites used the film design shown in Figure 3 for this work. Another film design was used for irradiations at the other two clinical sites. The second film design was a prototype design developed for the initial testing of the phantom.^17^ This prototype design did not have the circular and rectangular fiducial holes shown in Figure 3 and the slit, allowing the film pieces to interlock, terminated exactly at the center of each film piece; otherwise, the design was the same as shown in this figure. The film was digitized 3–7 days after irradiation using an Epson Perfection V750 Pro flatbed scanner (Epson America, Los Alamitos, CA). A cardboard template was used to align the film so that it was centered reproducibly on the scanner bed. The film images were saved as 24‐bit RGB TIF files at a resolution of 0.17 mm per pixel and processed using DoseLab. The RGB files were converted into optical density using the green channel. A third‐order polynomial fitted to 10 calibration points (*r* ^2^ = 0.999 for 0–22 Gy) was used to convert the optical density into dose. For the purposes of reducing the noise in the film images, each image was smoothed using a Wiener filter with a 5×5‐pixel window.

The measured dose distributions were evaluated using the dose intercomparison tools in DoseLab. Each film image was registered to a 10 × 10 cm (512 × 512 pixels) calculated dose plane, exported from Eclipse, which corresponded to the same position of the film when placed in the phantom for irradiation. The fiducial holes on the film were used to manually define translational and rotational registration points on the images. For the film pieces without fiducial holes, the slit running through each piece of film was used to define these points. In these cases, the point at which the slit terminates at the center of film was used as the translational registration point. The method of using the slit as a registration basis was validated previously.^17^ Following the manual registration, DoseLab optimized this alignment by applying additional shifts to the relative position of the calculated and measured dose distributions. These “auto‐registration shifts” were used as a metric for evaluating the targeting accuracy of the SRS treatments. Global gamma analyses using criteria of 3%/1 mm, 3%/0.5 mm, and 2%/1 mm were applied to the registered dose distributions. Each analysis was applied to a region of interest centered on the measured dose distribution measuring approximately 14 and 20 cm^2^ for the single and multitarget films, respectively. A dose threshold of 10% was applied in each case. The measured film dose was normalized to the calculated dose so that the gamma passing rate was optimized. The results of the gamma analysis were used as a metric for evaluating the dose modulation of the SRS treatments.

## 3 RESULTS AND DISCUSSION

### 3.1 Treatment planning

The maximum dose, D99 and PTV conformity index (CI) tolerances shown in Tables 1 and 2 were satisfied for every plan on each linac. Table 4 shows the distribution of the CI values organized by MLC and plan type. The distribution of the gradient index (GI), the ratio of the 50% and 100% dose volumes, for each plan is also shown for reference. The GI is not used for patient plan assessment at most of the clinical sites involved in this work; therefore, it was not included as a planning constraint. The GI values shown in Table 4 are consistent with published values in the literature.^34^ Both the GI and CI values obtained in this work are comparable for both types of MLC.

**TABLE 4:** Distribution of conformity index (CI) and gradient index (GI) values for the single target and multitarget treatment plans. The CI values have been calculated separately for each planning target volume (PTV). The GI has been calculated for the entire dose distribution associated with each plan

MLC type	Plan type	Average CI for 2‐cm PTV	Standard deviation CI for 2‐cm PTV	Average CI for 1‐cm PTV	Standard deviation CI for 1‐cm PTV	Average GI	Standard deviation GI
HDMLC	6‐MV FFF 1 PTV	1.09	0.08	NA	NA	2.94	0.30
	10‐MV FFF 1 PTV	1.12	0.08	NA	NA	3.09	0.15
	6‐MV FFF 2 PTV	1.09	0.06	1.24	0.20	3.41	0.73
	10‐MV FFF 2 PTV	1.09	0.04	1.21	0.18	3.62	0.58
Millennium	6‐MV FFF 1 PTV	1.08	0.06	NA	NA	3.37	0.70
	10‐MV FFF 1 PTV	1.10	0.07	NA	NA	3.50	0.80
	6‐MV FFF 2 PTV	1.11	0.07	1.17	0.09	3.80	0.90
	10‐MV FFF 2 PTV	1.09	0.08	1.18	0.17	4.05	0.54

Abbreviations: FFF, flattening filter free; HDMLC, high‐definition multileaf collimator; MLC, multileaf collimator; PTV, planning target volume.

### 3.2 Treatment delivery

The Winston–Lutz results showed a maximum difference between the center of the target ball and the center of the field of <1 mm across all six linacs. The mean value of the maximum difference was 0.79 mm with a standard deviation of 0.10 mm. These results provide a measurement of the coincidence of treatment isocenter with the CBCT‐aligned hidden target (brass ball) and satisfy published recommendations for SRS treatments.^9^, ^19^ These results show a larger difference than would be seen from a classic Winston–Lutz test,^8^ where there is no imaging component and the position of a target pointer can be iteratively adjusted to minimize the coincidence with isocenter. For comparison, a classic Winston–Lutz test was performed on an Edge linac back‐to‐back with the phantom Winston–Lutz procedure. The classic technique described above showed a maximum difference of 0.55 mm versus a maximum difference of 0.63 mm obtained with the phantom for the same combination of gantry, couch, and collimator angles. It should be noted that an off‐axis Winston–Lutz test was not included in this work; it is recommended that this test be included in routine QA for clinical sites delivering single‐isocenter, multitarget SRS plans.^35^

All chamber dose measurements performed across the six linacs agreed with the Eclipse dose to within 3%. Table 5 shows the mean and standard deviation of the dose difference for the single‐target plans; there is no significant difference between energy or MLC type.

**TABLE 5:** Absolute dosimetry results for the single‐target plans. The average and standard deviation of the dose difference between the pinpoint chamber measurements and the calculated dose from Eclipse is shown for each energy and multileaf collimator (MLC) type

Single target plan type	Average % dose difference between chamber measurement and calcuation	Standard deviation of % dose difference between chamber measurement and calculation
6‐MV FFF	–0.1	2.1
10‐MV FFF	–0.6	1.8
Millennium MLC	0.1	2.3
HDMLC	–0.8	1.5

Abbreviations: FFF, flattening filter free; HDMLC, high‐definition multileaf collimator; MLC, multileaf collimator.

**FIGURE 4:**
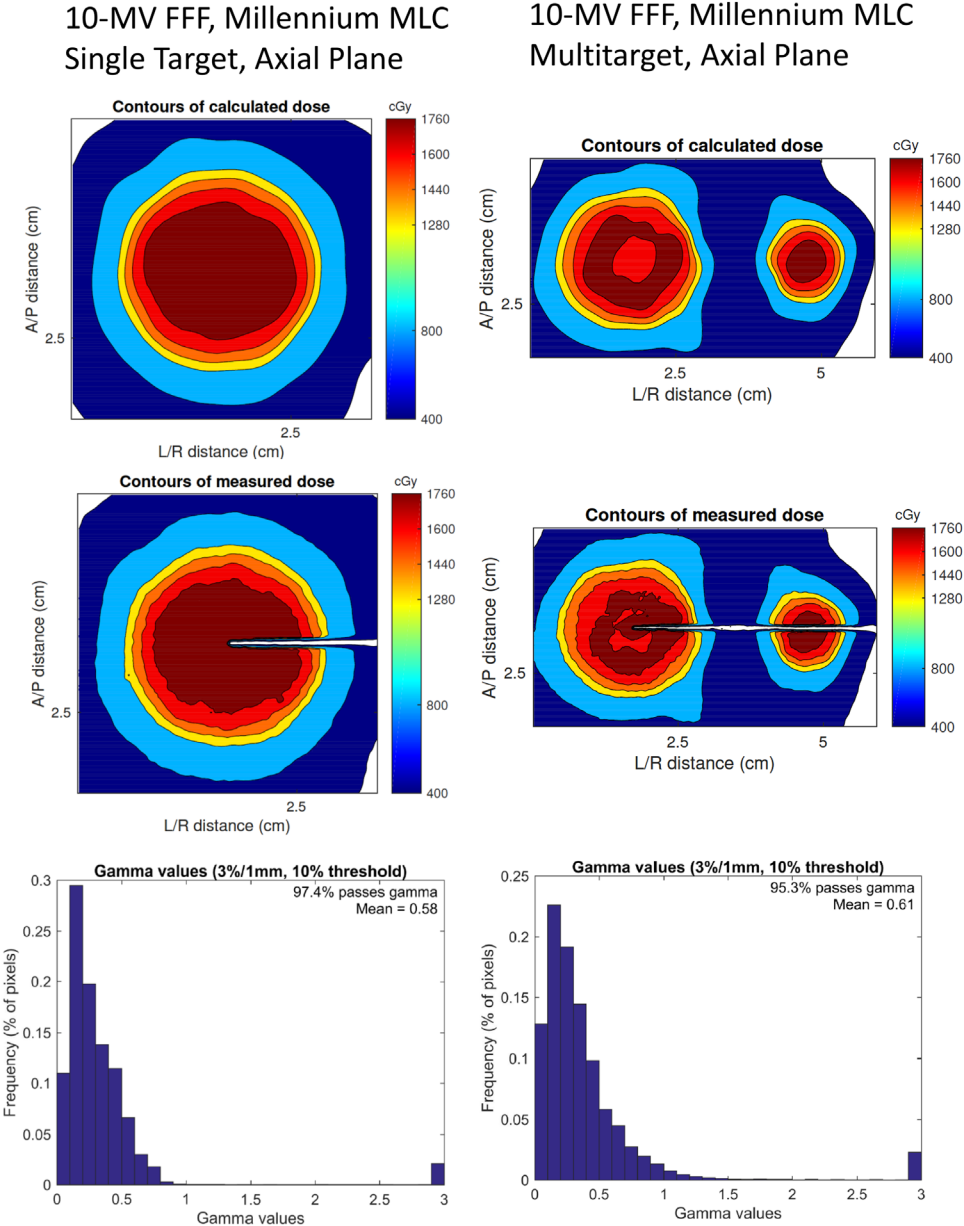
DoseLab analysis for film irradiations at 10‐MV FFF on a TrueBeam with a Millennium multileaf collimator (MLC). The analysis shown here was performed using gamma criteria of 3%/1 mm. Results in the axial plane for single and multitarget plans are shown on the left and right, respectively

**FIGURE 5:**
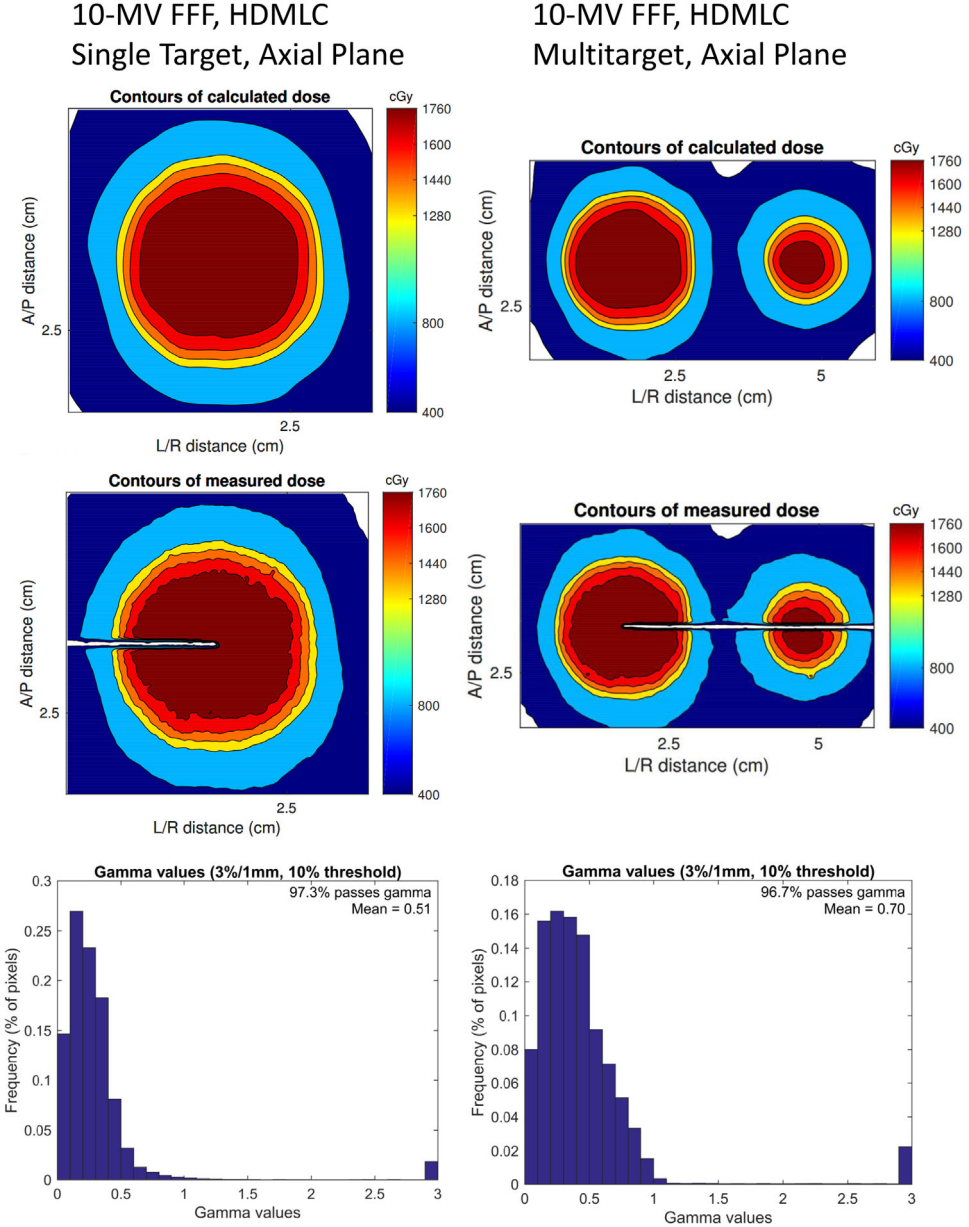
DoseLab analysis for film irradiations at 10‐MV FFF on a TrueBeam with a high‐definition multileaf collimator (HDMLC). The analysis shown here was performed using gamma criteria of 3%/1 mm. Results in the axial plane for single and multitarget plans are shown on the left and right, respectively

Table 6 shows the gamma‐passing statistics for the irradiated film. Data are shown for criteria of 3%/1 mm, 3%/0.5 mm, and 2%/1 mm for separate categories of treatment plan. These gamma results were obtained following auto‐registration shifts ≤ 1 mm in any direction. The results illustrate no significant difference in the pass rate between MLC type, target number, or energy. All the film data show gamma passing > 92% for criteria of 3%/1 mm. These results are consistent with patient‐specific QA data reported by Wen et al.^22^ who obtained an average passing of 95.0±4.2% with the same criteria for 83 SRS and stereotactic body radiation therapy (SBRT) plans measured with EBT3 film. The passing rate is scattered over a larger range for criteria of 3%/0.5 mm and 2%/1 mm, but the average is still > 90% in both cases. Tables 7 and 8 show the film results taken from TrueBeam machines with Millennium MLC and HDMLC, respectively. For the same two machines, Figures 4 and 5 give examples of the comparison of the measured and calculated dose distributions for 10‐MV FFF single‐target and multitarget plans. These data are representative of the data taken across all six linacs.

**TABLE 6:** Gamma passing statistics for irradiated film organized by plan type. Film results are shown for three sets of gamma criteria for the single target (1 PTV), multitarget (2 PTV), high‐definition multileaf collimator (HDMLC), Millennium multileaf collimator (MLC), 6‐ and 10‐MV flattening filter‐free (FFF) plans. A 10% dose threshold was applied for each set of criteria

Plan type	Gamma passing (3%/1 mm)	Gamma passing (3%/0.5 mm)	Gamma passing (2%/1 mm)
1 PTV	Max = 99.9%	Max = 99.3%	Max = 99.0%
	Min = 92.2%	Min = 83.3%	Min = 71.1%
	Ave = 96.5%, STDEV = 2.4%	Ave = 94.4%, STDEV = 3.8%	Ave = 93.4%, STDEV = 5.8%
2 PTV	Max = 98.8%	Max = 98.1%	Max = 97.7%
	Min = 92.2%	Min = 79.9%	Min = 85.6%
	Ave = 95.6%, STDEV = 2.2%	Ave = 91.2%, STDEV = 4.9%	Ave = 92.6%, STDEV = 3.3%
HDMLC	Max = 99.9%	Max = 99.3%	Max = 99.0%
	Min = 92.2%	Min = 83.3%	Min = 71.1%
	Ave = 96.2%, STDEV = 2.3%	Ave = 93.6, STDEV = 4.5%	Ave = 92.9%, STDEV = 5.8%
Millennium MLC	Max = 99.8%	Max = 99.0%	Max = 98.5%
	Min = 92.2%	Min = 79.9%	Min = 85.6%
	Ave = 95.8%, STDEV = 2.5%	Ave = 91.9%, STDEV = 4.7%	Ave = 93.1%, STDEV = 3.1%
6‐MV FFF	Max = 99.9%	Max = 99.0%	Max = 99.0%
	Min = 92.2%	Min = 79.9%	Min = 71.1%
	Ave = 96.0%, STDEV = 2.5%	Ave = 92.8%, STDEV = 4.8%	Ave = 92.6%, STDEV = 5.7%
10‐MV FFF	Max = 99.8%	Max = 99.3%	Max = 98.2%
	Min = 92.2%	Min = 84.6%	Min = 85.6%
	Ave = 96.1%, STDEV = 2.3%	Ave = 92.8%, STDEV = 4.5%	Ave = 93.5%, STDEV = 3.1%

Abbreviations: Ave, average; Max, maximum; Min, minimum; PTV, planning target volume; STDEV, standard deviation.

**TABLE 7:** Example of film results obtained from a TrueBeam with a Millennium multileaf collimator (MLC). The gamma analysis results and the auto‐registration shifts determined by DoseLab are shown for each irradiated film. The auto‐registration shifts were calculated in the anterior‐posterior (A‐P), left‐right (L‐R), and superior‐inferior (S‐I) directions

MLC Type	Plan type	Technique	Film plane	% Gamma passing (3%/1 mm)	A‐P shift (mm)	L‐R shift (mm)	S‐I shift (mm)
Millennium	6‐MV FFF 1 PTV	4‐field RapidArc	Axial	97.3	1.0	0.4	NA
			Coronal	97.0	NA	0.2	0.0
	10‐MV FFF 1 PTV	4‐field RapidArc	Axial	97.4	0.4	0.0	NA
			Coronal	95.1	NA	0.2	0.2
	6‐MV FFF 2 PTV	4‐field RapidArc	Axial	93.6	0.4	0.0	NA
			Coronal	96.0	NA	0.4	0.0
	10‐MV FFF 2 PTV	4‐field RapidArc	Axial	95.3	0.6	0.2	NA
			Coronal	96.5	NA	0.2	‐0.6

**TABLE 8:** Example of film results obtained from a TrueBeam with a high‐definition multileaf collimator (HDMLC). The gamma analysis results and the auto‐registration shifts determined by DoseLab are shown for each irradiated film. The auto‐registration shifts were calculated in the anterior‐posterior (A‐P), left‐right (L‐R), and superior‐inferior (S‐I) directions

MLC type	Plan type	Technique	Film plane	% Gamma passing (3%/1 mm)	A‐P shift (mm)	L‐R shift (mm)	S‐I shift (mm)
HDMLC	6‐MV FFF 1 PTV	4‐field RapidArc	Axial	96.6	0.2	0.6	NA
			Coronal	96.8	NA	0.6	–0.6
	10‐MV FFF 1 PTV	4‐field RapidArc	Axial	97.3	0.4	0.6	NA
			Coronal	97.1	NA	0.6	–0.8
	6‐MV FFF 2 PTV	4‐field RapidArc	Axial	95.0	0.0	0.0	NA
			Coronal	98.4	NA	–0.2	0.4
	10‐MV FFF 2 PTV	4‐field RapidArc	Axial	96.7	0.2	0.0	NA
			Coronal	98.4	NA	0.2	0.4

### 3.3 Data outliers

Two treatment plans, delivered on different machines, produced film results that were inconsistent with the film data discussed in Section 3.2. The first plan, a 6‐MV FFF multitarget plan, showed a gamma passing rate of 85.4% in the coronal plane for criteria of 3%/1 mm. This passing rate is approximately three standard deviations below the minimum passing rate for multitarget plans shown in Table 6. Subsequent plan QA performed with a MapCheck2 device (Sun Nuclear, Melbourne, Fl) showed a gamma passing rate of 81.1% for the same criteria. Although the MapCheck2 is not ideal for SRS measurements due to its detector spacing,^36^ patient‐specific QA with this device consistently show > 95% passing at this clinical site for SRS plans with 3%/1 mm gamma criteria. This plan would not be acceptable for local SRS treatments; therefore, the data were rejected and not considered for this work. The modulation factor for this plan (3.9) was high compared to the other plans developed for the same linac (range = 2.9 – 3.5) and this is a possible cause of the poor QA results. The second plan, a 10‐MV FFF single‐target plan delivered using DCA, produced a gamma passing rate of > 90% for all three sets of gamma criteria, but showed an auto‐registration shift of 1.2 mm in the anterior direction.^18^ This was the only case (out of 48 films) that showed a shift > 1 mm. The auto‐registration shifts provide a measurement of the treatment delivery accuracy; they are heavily dependent on user‐determined CBCT alignment of the phantom and are also affected by beam steering within the linac. A similar anterior shift of 1.0 mm for the 10‐MV FFF multitarget plan on the same machine and smaller shifts (≤0.6 mm) for the plans at 6‐MV FFF suggests a systematic effect at 10‐MV FFF. Regardless, the 1.2 mm shift does not satisfy the geometric tolerance of 1 mm for SRS treatments in the AAPM Medical Physics Practice Guideline (MPPG) 9.a.^9^ The results associated with this plan were rejected and not considered for this work.

## 4 CONCLUSIONS

Dosimetric and geometric end‐to‐end data for MLC‐based, SRS treatments have been successfully acquired for six TrueBeam and Edge linear accelerators using the Varian SRS phantom. Commissioning criteria shown in Table 9 are recommended for end‐to‐end SRS measurements performed with this phantom on these machines. These criteria are based on the distribution of data acquired in this work and the boundary conditions imposed by the AAPM recommendations for SRS end‐to‐end measurements.^9^, ^19^ The chosen gamma criteria of 3%/1 mm and passing threshold of 90% reflect the narrow distribution of film results. These parameters are tighter than those utilized for film analysis with the IROC SRS phantom^11^, ^21^ and are consistent with recommendations published by Xia et al. for patient‐specific SRS QA.^24^ Although the absolute dose measurements consistently agreed to within 3% of the calculated dose in this work, MPPG 9.a. recommends a more lenient ±5% difference between measured and calculated dose differences.^9^ The absolute dosimetry in this work was performed with a single chamber; a higher threshold of ±5% is adopted to account for chamber‐to‐chamber calibration uncertainty^30^ and for consistency with MPPG 9.a. It should be noted that these recommendations are based on data exclusively obtained using a single‐vendor environment comprised of Varian linacs, ARIA, Eclipse, and DoseLab software. The recommended gamma criteria and passing threshold could be applied to film results obtained from software other than DoseLab; however, the auto‐registration shifts, which provide a measurement of the treatment delivery accuracy, are only applicable to film results obtained from this software application.

**TABLE 9:** Recommended commissioning criteria for end‐to‐end stereotactic radiosurgery (SRS) measurements performed with the Varian SRS phantom on TrueBeam and Edge machines

Phantom test	Tolerance
Winston–Lutz test following hidden target (brass ball) alignment with CBCT	Target coincidence <1 mm
Absolute dose measurement with ion chamber	Agreement with calculation ±5%
EBT3 film irradiation	Gamma passing > 90% for 3% and 1 mm with DoseLab auto‐registration shifts ≤ 1 mm (all film planes)

Abbreviation: CBCT, cone‐beam computed tomography.

## CONFLICT OF INTEREST

This work received financial support from Varian Medical Systems.

## AUTHOR CONTRIBUTIONS

All authors made significant contributions to this work. Thomas A. D. Brown is the primary author and was responsible for writing the paper and performing all the data analysis described therein. The first four authors from Northwest Medical Physics Center and the authors from Varian were responsible for designing the end‐to‐end procedure described in this work. All the authors from Northwest Medical Physics Center were responsible for collecting end‐to‐end data. All authors have reviewed the paper and were given the opportunity to provide edits prior to submission.

## Data Availability

Research data are not shared.
